# Adjuvant therapeutic effects of moxibustion on COVID-19: A protocol for systematic review and meta-analysis

**DOI:** 10.1097/MD.0000000000023198

**Published:** 2020-11-13

**Authors:** Zhi-lei Wang, Juan Zhang, Dong-qing Du, Feng-jun Ma, Xiao Yan, Chen Chen, Yu-xia Ma

**Affiliations:** a Department of Acupuncture and Massage, Shandong University of Traditional Chinese Medicine, Shandong, Jinan; b Shandong College of Traditional Chinese Medicine, Shandong, Yantai, China.

**Keywords:** moxibustion, COVID-19, systematic review, meta-analysis, protocol

## Abstract

**Background:**

COVID-9 has become a global pandemic with severe health issues around the world. However, there is still no effective drug to treat the disease, and many studies have shown that moxibustion plays a positive role in adjuvant treatment of COVID-19. Therefore, this meta-analysis is designed to evaluate the efficacy of moxibustion for COVID-19.

**Methods:**

The relevant randomized controlled trials will be systematically retrieved from the electronic database, including PubMed, Embase, Cochrane Clinical Trials Database, Web of Science, and China National Knowledge Infrastructure, without restrictions on publication status and language. Two reviewers will independently review all included studies and assess the risk of bias. Two reviewers will independently extract data from the included studies based on a pre-designed standardized form. Any disagreements will be resolved by consensus. The meta-analysis will be performed with RevMan (V5.3.5) software.

**Result:**

The results of this study will be published in a peer-reviewed journal.

**Conclusion:**

This ongoing meta-analysis will provide up-to-date evidence of the efficacy of moxibustion for patients with COVID-19.

**Registration:**

The meta-analysis has been prospectively registered in PROSPERO (CRD42020211910).

## 1 Introduction

COVID-19 is an infection caused by the SARS-CoV-2 virus, initially identified in the city of Wuhan, China, in December 2019. Since then, the virus has spread to the continents, causing a major pandemic.^[1]^ COVID-9 has become a global pandemic with severe health issues around the world.^[2]^ Epidemiological investigations have shown that main symptoms of COVID-19 are fever, dry cough, and fatigue, with a small number of patients suffering from nasal congestion, sore throat, runny nose, myalgia, and diarrhea.^[3]^ Moxibustion, as a common treatment of traditional Chinese medicine (TCM), played the auxiliary role of TCM in the treatment of Severe Acute Respiratory Syndrome (SARS) in 2003.^[4]^ Therefore, in China, where COVID-19 was first popular, moxibustion is also used as an adjuvant therapy for COVID-19. Although many studies have shown that moxibustion plays a positive role in adjuvant treatment of COVID-19,^[5–8]^ there is no systematic review on the efficacy of moxibustion in the treatment of COVID-19. Therefore, the study we are going to conduct is a systematic review and meta-analysis of the efficacy of moxibustion in the treatment of COVID-19.

## 2 Methods

We will conduct the systematic review according to preferred reporting items for systematic review and meta-analysis protocols 2015 statement.^[9]^ This meta-analysis has been registered in PROSPERO (CRD42020211910) for quality control.

### 2.1 Study inclusion criteria

#### 2.1.1 Types of studies

Randomized controlled trials of COVID-19 treated with moxibustion will be included and pooled in the assessment. Non randomized controlled clinical studies and clinical case reports will be reported.

#### 2.1.2 Participants

Patients diagnosed with COVID-19, without gender, race, educational status, and age restrictions.

#### 2.1.3 Types of interventions

The intervention of the experimental group should include moxibustion, which can be carried out alone, or combined with other kinds of therapies. The control group will receive any kind of treatment without moxibustion (Western medicine, placebo or regular treatment).

#### 2.1.4 Outcomes

In this meta-analysis, the primary outcomes assessed is the total clinical response rate, and the diagnostic criteria included Cured: the disappearance of the main symptoms, the normal body temperature, the disappearance of lung rales, the re-examination of chest X-ray showed the absorption of lung lesions, and the return of white blood cell count to normal; Effective: the main symptoms were relieved, lung rhombus was improved, chest X-ray showed that the lung lesions were not completely absorbed, and the white blood cell count was improved; Invalid: no improvement or aggravation of symptoms, signs, chest X-ray examination and abnormal white blood cell count. Total clinical effective rate = (number of cured cases + number of effective cases)/total number of cases 100%; Secondary outcome measures: antipyretic time, cough duration, rhombus disappearance time, imaging transition time, serum C-reactive protein level after treatment.^[10]^

### 2.2 Search strategy

Two independent reviewers (ZLW and JZ) will search PubMed, Embase, Cochrane Clinical Trials Database, Web of Science, and China National Knowledge Infrastructure. The search strategy in PubMed is as follows:

#1 “moxibustion” [Title/Abstract] OR “moxa” [Title/Abstract] OR “acupoint” [Title/Abstract] OR “TCM”[Title/Abstract] OR “ Traditional Chinese medicine” [Title/Abstract]
#2 “COVID-19” [MeSH Terms] OR “2019 novel coronavirus infection” [Title/Abstract] OR “COVID19” [Title/Abstract] OR “coronavirus disease 2019” [Title/Abstract] OR“coronavirus disease-19” [Title/Abstract] OR “2019-nCoV disease” [Title/Abstract] OR “2019 novel coronavirus disease” [Title/Abstract] OR “2019-nCoV infection”[Title/Abstract] OR “ 2019-nCoV ” [Title/Abstract] OR “ nCoV ” [Title/Abstract] OR “ SARS-CoV∗ ” [Title/Abstract] OR “ SARSCov2 ” [Title/Abstract] OR “SARS-CoV ” [Title/Abstract] OR “ 2019 coronavirus ” [Title/Abstract]
#3 “randomized controlled trial” [Publication Type] OR “controlled clinical trial” [Publication Type] OR “Single-Blind Method” [Text Word] OR “Double-Blind Method”[Text Word] OR “random allocation” [Text Word] OR “allocation” [Text Word] OR “ randomized controlled trials” [Text Word]
#4 #1 AND #2 AND #3

### 2.3 Data collection and management

#### 2.3.1 Study selection

**Figure 1:**
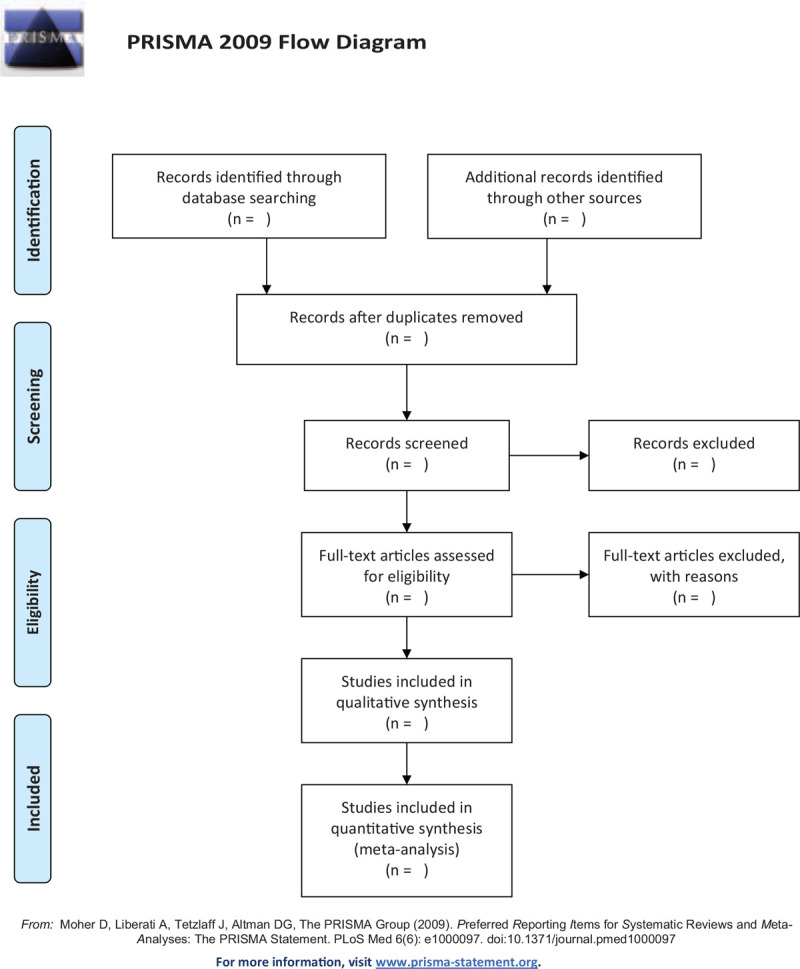
The PRISMA flow diagram. PRISMA = preferred reporting items for systematic review and meta-analysis.

After literature retrieval, the literature records will be imported into EndNoteX9 software for management. We will first exclude duplication, and then 2 authors (XY and JFM) will independently evaluate the literature search for relevant abstracts and titles based on pre-defined inclusion criteria. A third author will arbitrate any disagreement between the 2 authors. If the full text is not available, we will try to contact the appropriate author. The selection process will be presented in a preferred reporting items for systematic review and meta-analysis protocols flow diagram (Fig. 1).

#### 2.3.2 Data extraction

Two authors (CC and DQD) will select the literature and extract the data according to the retrieval strategy. The title of the study, the name of the first author, the year of publication, the journal; the participants’ information: gender, age, study design, sample size, diagnostic criteria, intervention and outcome indicators will be independently extracted from the included study by the 2 reviewers in a standardized form, they will also cross-check the results, and the differences will be resolved by recommending a third author (ZL W).

#### 2.3.3 Dealing with missing data

We will attempt to obtain missing data by contacting the corresponding author. If it fails, we will analyze it based on available data.

#### 2.3.4 Risk of bias assessment

Through the RevMan v5.3 software built-in risk bias assessment tool provided by Cochrane Collaboration Network; the risk bias assessment will be carried out for the included study. The evaluation contents include random sequence generation, allocation concealment, blinding of participants and personnel, blinding of outcome assessment, incomplete outcome data, selective reporting and other risk bias. According to the performance of the included literature in the above evaluation items, if all key domains are graded as low risk, this study will be graded as low risk; if 1 or more key domains are graded as high risk, this study will be graded as high risk; if 1 or more key domains are graded as unclear risk, this study will be graded as unclear risk.

#### 2.3.5 Assessment of quality of evidence

We will use Grading of Recommendations Assessment, Development and Evaluation (GRADE) system for rating overall quality of evidence supporting selected primary and secondary outcomes. This work will be completed by 2 researchers (ZLW and JZ). If there are different opinions, they can be solved through discussion.

#### 2.3.6 Date synthesis

Data synthesis will be performed with RevMan v5.3. For dichotomous data, we will calculate the date and present it by the relative risks with 95% confidence intervals. For continuous data, we will calculate the effect size using the mean differences or standardized mean difference with 95% confidence intervals. Heterogeneity will be assessed by *I*^2^. It is acknowledged that *I*^2^ < 25% indicates negligible heterogeneity, 25%≤*I*^2^ < 50% indicates mild heterogeneity, 50%≤*I*^2^ < 75% moderate heterogeneity, and *I*^2^ ≥75% high heterogeneity. Fixed- and random-effects models will be used when *I*^2^ ≤50% and *I*^2^ > 50%, respectively. The results will be presented as forest plots.

#### 2.3.7 Subgroups and sensitivity analysis

If data are available, we will conduct a subgroup analysis according to different interventions, controls, durations of treatment, and outcome measures. We will conduct a sensitivity analysis to assess the robustness of the meta-analysis results. Sensitivity analysis is used to analyze research quality, method elements, publishing type, and publishing language and so on. If there is a high degree of heterogeneity, meta-analysis will be repeated to exclude low-quality or small sample studies.

#### 2.3.8 Assessment of publication biases

The funnel plots in RevMan V.5.3 will be used to detect publication bias if >10 studies are included in the meta-analysis.

#### 2.3.9 Ethics and dissemination

There is no need for this study to require ethical approval and informed consent because it is based on published literatures. The results of this meta-analysis will be submitted to a peer-reviewed journal for publication and information sharing.

## 3 Discussion

Due to the lack of specific drugs, COVID-19 in many countries around the world has not been effectively controlled since the outbreak in 2019. In China, moxibustion has played a role in preventing and treating plague since ancient times.^[11]^ Previous studies have shown that moxibustion plays a good auxiliary role in the treatment of common pneumonia and SARS.^[4,12]^ At the beginning of the outbreak in China, the World Federation of Acupuncture and moxibustion Societies issued guidelines on moxibustion intervention in COVID-19.^[13]^ It is recommended to use moxibustion as an adjuvant treatment of COVID-19. Moxibustion has contributed to the prevention and treatment of COVID-19 in China. At present, China has achieved remarkable results in epidemic prevention, but the situation in other countries is still grim. Therefore, we will systematically review the role of moxibustion in the adjuvant therapy of COVID-19 and provide reference for the global fight against the epidemic.

## Author contributions

ZLW, JZ, and YXM contributed to conceiving and designing the experiments; DQD, FJM, XY and CC gave some suggestions for modification. All authors have read and approved the manuscript.

**Data curation:** Juan Zhang, Xiao Yan.

**Funding acquisition:** Yu-xia Ma.

**Methodology:** Dong-qing Du, Feng-jun Ma.

**Software:** Dong-qing Du, Feng-jun Ma, Xiao Yan, Chen Chen.

**Supervision:** Yu-xia Ma.

**Visualization:** Chen Chen.

**Writing – original draft:** Zhi-lei Wang.

**Writing – review & editing:** Zhi-lei Wang, Yu-xia Ma.

## References

[1] Lemos G Araújo D de Lima F Human anatomy education and management of anatomic specimens during and after COVID-19 pandemic: ethical, legal, and biosafety aspects. Ann Anat 1516;2020:08. 10.1016/j.aanat.2020.151608 PMC7532794 33022405

[2] Esmaeilzadeh A Elahi R Immunobiology and immunotherapy of COVID-19: a clinically updated overview. J Cell Physiol 2020. 10.1002/jcp.30076 PMC7675260 33022076

[3] Adhikari S Meng S Wu Y Epidemiology, causes, clinical manifestation and diagnosis, prevention and control of coronavirus disease (COVID-19) during the early outbreak period: a scoping review. Infect Dis Poverty 2020;9:29. 32183901 10.1186/s40249-020-00646-x PMC7079521

[4] Z H L Y L B Nine cases of the chronic stage of SARS treated by moxibustion. Chinese Acupuncture & Moxibustion 2003;23:564–5.

[5] D.S W Q G L Observation on the therapeutic effect of syndrome differentiation and moxibustion on diarrhea in 36 patients with COVID-19 in Fangdang Hospital. Chinese Acupuncture & Moxibustion 2020;40:690–2.

[6] H X X D Q Q Clinical observation of heat-sensitive moxibustion treatment for coronavirus disease 2019. Chinese Acupuncture & Moxibustion 2020;40:576–80. 32538004 10.13703/j.0255-2930.20200312-k0003

[7] C Y S T S Q An overall analysis of non - drug intervention programs for COVID-19. Journal of Shaanxi University of Chinese Medicine 2020;43:19–25.

[8] Huang X Xie D Qiu Q 42 cases of coronavirus disease 2019 of the ordinary type with the adjuvant treatment of heat-sensitive moxibustion 42. World journal of acupuncture-moxibustion 2020;30:163–6. 32837110 10.1016/j.wjam.2020.08.003 PMC7428449

[9] Shamseer L Moher D Clarke M Preferred reporting items for systematic review and meta-analysis protocols (PRISMA-P) 2015: elaboration and explanation. BMJ (Clinical research ed) 2015;350:g7647. 10.1136/bmj.g7647 25555855

[10] Zhang Q Xu X Sun S Efficacy of acupuncture and moxibustion in adjuvant treatment of patients with novel coronavirus disease 2019 (COVID-19): a protocol for systematic review and meta analysis. Medicine 2020;99:e21039. 32664113 10.1097/MD.0000000000021039 PMC7360278

[11] Z J X Y The enlightenment of Tang Dynasty physician Sun Simiao's epidemic prevention method of external use of traditional Chinese medicine on the prevention of infectious atypical pneumonia. Chinese Journal of Clinical Rehabilitation 2003;7:3772.

[12] S T C Z Effects of moxa-moxibustion plus infrared illumination on community acquired pneumonia and immune function. Clinical Journal of Chinese Medicine 2020;12:30–2.

[13] Liu W Guo S Wang F guidance for acupuncture and moxibustion interventions on COVID-19Understanding of (Second edition) issued by CAAM. World journal of acupuncture-moxibustion 2020;30:1–4. 32292259 10.1016/j.wjam.2020.03.005 PMC7118592

